# Basic psychological needs in gambling and gaming problems

**DOI:** 10.1016/j.abrep.2022.100445

**Published:** 2022-07-03

**Authors:** Ilkka Vuorinen, Iina Savolainen, Heli Hagfors, Atte Oksanen

**Affiliations:** Tampere University, Faculty of Social Sciences, Tampere University, 33014 Tampere, Finland

**Keywords:** Gambling, Gaming, Basic psychological needs, Mental health

## Abstract

**Background and Aims:**

Studies on self-determination theory (SDT) have placed satisfaction of basic psychological needs (autonomy, relatedness, and competence) at the core of well-being, whereas frustration of these needs results in multiple potentially unhealthy mental and behavioral patterns. This study analyzed how need satisfaction and frustration relate to the severity of gambling and gaming problems.

**Methods:**

A survey study with 18–75-year-old Finnish participants (*N* = 1530; 50.33% male) was conducted in April 2021. Basic psychological needs were measured with the Basic Psychological Need Satisfaction and Frustration Scale, mental health issues with the five-item Mental Health Inventory, gambling problems with the Problem Gambling Severity Index, and gaming problems with the Internet Gaming Disorder Test. Zero-inflated negative binomial analyses were conducted to examine how satisfaction and frustration of basic psychological needs, together with mental health issues, are associated with gaming and gambling problems.

**Results:**

Mental health issues were associated with gambling and gaming problems, but this association became nonsignificant when basic psychological needs were added to the model. However, better mental health still was associated with the absence of gaming problems. While need satisfaction had no association with the absence of gaming or gambling problems, need frustration was associated with increases in the severity of both gaming and gambling problems.

**Discussion:**

Frustration of basic psychological needs for autonomy, relatedness, and competence is associated with both gambling and gaming problems and should be considered when developing treatment and support for those who experience such problems.

## Highlights

• Need frustration was related to the severity of gambling and gaming problems.
• Need satisfaction was not related to the absence of gambling or gaming problems.
• Higher mental health problems were related to the risk of any gaming problems.

## 1 Introduction

Gambling and digital gaming have become increasingly popular forms of entertainment as technological advancements such as personal computers, gaming consoles, the Internet, and smartphones have brought them closer and made them more accessible. In Finland, 29% of the population gamble, and 28% play digital games at least once a week (Salonen et al., 2020). For most people, these games may represent personal and social rewards or simply a chance of winning (Binde, 2013, Boyle et al., 2011), but the risks for personal, social, and financial problems increase along with increased gambling and gaming (Buono et al., 2020, Castrén et al., 2018, Jeffrey et al., 2019).

As with other potentially excessive behaviors, nationwide prevalence estimates for more severe gambling and gaming problems are usually low. The most recent national prevalence rates in Finland were 0.7% for “probable pathological gambling” (Problem Gambling Severity Index [PGSI] ≥ 8) and 1.3% for “problematic gaming” (Gaming Addiction Scale [GAS-7] ≥ 4), although gambling and gaming problems affect a larger proportion of the population (Salonen et al., 2020). It is also more likely for young men to belong to high-risk groups (Dowling et al., 2017, Macur and Pontes, 2021). In psychiatry, gambling and gaming problems are thought to include preoccupation, attempts to escape adverse mood states, difficulty to control the activity, deception, and jeopardized or lost social opportunities and relationships (American Psychiatric Association, 2013, Petry et al., 2014). Comorbidity with mental health issues such as depression, anxiety, excessive substance use, and impulse-control disorders is common in severe gambling and gaming problems (APA, 2013; Kessler et al., 2008, Kuss and Griffiths, 2012, Petry et al., 2005). However, estimates of prevalence and comorbidity vary depending on factors like study design (Dowling et al., 2015, Ferguson et al., 2011).

One possible yet relatively overlooked theoretical perspective regarding gambling and gaming problems is the perspective of basic psychological needs. According to the Self-Determination Theory (SDT; Ryan and Deci, 2000, Ryan and Deci, 2017), there are three interdependent basic psychological needs—autonomy, relatedness, and competence—that must be satisfied for a person to flourish and function in a healthy way. *Autonomy* refers to the need to self-regulate one’s experiences and actions, whereas the need to feel socially connected and engaged is the core element of *relatedness*. Finally, *competence* depicts the need to feel optimally efficient and skillful in one’s efforts. Neurologically, these needs have been described to associate particularly with reward processing networks and insular activity, which are present in various motivation-based and emotional tasks (Reeve & Lee, 2019), including addictive behaviors (Heilig et al., 2016, Volkow and Boyle, 2018). It can therefore be assumed that basic psychological needs are also involved in the trajectories of gambling and gaming behaviors.

Even though satisfaction of basic psychological needs is a necessary requirement for a person to flourish, social contexts may not always support the fulfilment of these needs, which can lead to various maladaptive substitute behaviors (Deci & Ryan, 2000). Basic psychological needs can also be actively thwarted, and further developments of SDT have suggested that the resulting frustration might not be adequately captured by looking merely at low scores of need satisfaction (e.g., it is different to feel rejected than dissatisfied in one’s love affairs), making the constructs of need satisfaction and need frustration asymmetrical (Bartholomew et al., 2011, Bartholomew et al., 2011, Costa et al., 2015, Vansteenkiste and Ryan, 2013). Vansteenkiste and Ryan (2013) also pointed out that people with chronic need frustration are more likely to pursue external indicators of worth such as popularity or wealth, behave rigidly, lack self-control, and display oppositional defiance, which promote negative cycles of further frustration, maladaptation, and ill-being.

In the field of addiction research, studies have mostly applied a broader SDT framework to study motivation in treatment outcomes (e.g. Chan et al., 2019, Kennedy and Gregoire, 2009, Kushnir et al., 2016), while many other studies have taken an approach toward gambling or gaming problems using concepts that are comparable to basic psychological needs. Concerning gambling problems, mediation analyses by Rodriguez et al. (2015) suggest that autonomously motivated individuals are less likely to try to win back gambling losses and gamble as an escape which in turn protects them against gambling problems. Studies have also found that strong social ties can be a protective factor against gambling problems (Nordmyr et al., 2016, Oksanen et al., 2021). Furthermore, Bergen et al. (2014) have demonstrated that gambling can enhance a sense of self-control that those who experience more severe gambling problems tend to lack in their everyday life.

Some recent studies have brought attention specifically to the satisfaction and frustration of basic psychological needs in the context of gambling or gaming problems. For example, Mills et al. (2021) found that general lack of motivation and motivation that is oriented towards external approval were associated with gambling problems through frustration of basic psychological needs. One study on elderly adults who gamble suggests that basic psychological need satisfaction decreases their risk for gambling problems (Dennis et al., 2017) and a study on Chinese adults shows that basic psychological need satisfaction predicts adherence to responsible gambling practices indirectly through flourishing (Tong et al., 2022).

Regarding digital gaming, earlier studies grounded in SDT have provided evidence that motivation and engagement towards playing a game is influenced by need satisfaction-supportive features of the game (Peng et al., 2012, Przybylski et al., 2010, Ryan et al., 2006). As for gaming problems, a study by Tóth-Király et al. (2019) suggests that general need satisfaction is a protective factor and need frustration a risk factor for obsessive and maladaptive passion towards gaming. Other studies have found that need frustration in daily life and need satisfaction in digital games are associated with the severity of gaming problems (Allen and Anderson, 2018, Bender and Gentile, 2020, Mills et al., 2018). Furthermore, it is possible that need frustration increases gaming problems at least partially by lowering self-control (Mills & Allen, 2020) and self-esteem as well as increasing depressive symptoms (Scerri et al., 2019). Hence, it can be stated that satisfaction and frustration of basic psychological needs for autonomy, relatedness and competence act an important part in problems related to gambling and gaming.

Although previous research has offered insight into how satisfaction or frustration of basic psychological needs relate to addictive behaviors, there is a lack of research investigating the role of these needs in gambling and gaming problems while accounting for comorbidity with mental health issues. In the present study, we analyze the association of basic psychological needs and gambling and gaming problems while controlling for mental health issues, age, and gender. Based on the available literature, our hypotheses are:

H1: Higher need frustration is associated with higher gambling and gaming problems.

H2: Higher need satisfaction is associated with the absence of gambling and gaming problems.

H3: Mental health issues interact with need satisfaction and frustration.

## 2 Material and methods

### 2.1 Participants

A cross-sectional survey was collected online from 18 to 75-year-old Finnish panel volunteers (*n* = 1530) by a data provider company, Norstat Finland, in April 2021. The participants were contacted randomly via email and the provider’s mobile application. The mean age of the participants was 46.7 years (*SD* = 16.4 years); 50.3% of them were men, 49.4% were women, and 0.3% identified their gender as “other”. The survey sample was designed to be nationally representative, and it covered all Finnish-speaking regions of mainland Finland. The response rate of the survey was 34.6%.

Before the start of the data collection, The Academic Ethics Committee of Tampere region gave their approval for the study. The participants gave their consent for participation by completing the full survey. The research group worked only with anonymized data provided by Norstat. Data quality checks were conducted to detect and evaluate possible biases in responses following a pre-established protocol. During a review of open-ended feedback, these checks revealed a few biased response patterns, which led to the exclusion of three participants; 1530 were left for the final data.

### 2.2 Measures

Gambling problems were assessed with the Problem Gambling Severity Index (PGSI; Ferris & Wynne, 2001), which is a commonly used survey tool for measuring gambling-related problems (Grigsby, 2020) and has been used previously in Finland (Edgren et al., 2016, Salonen et al., 2020). The PGSI consists of nine items that measure, for example, increasing costs of gambling, need for excitement, chasing money lost in gambling, negative social and economic feedback, and guilt. In this study, we asked the participants to evaluate their experiences during the last six months. The response choices were 0 (*never*), 1 (*sometimes*), 2 (*most of the time*), and 3 (*almost always*). Considering the dissenting views for the classification and threshold scores for problem gambling (Currie et al., 2010, Stone et al., 2014), we left the scale in its count form. The scale had excellent internal consistency (McDonald’s omega, ω = 0.95). However, 72.09% of the participants had not experienced any gambling problems within the last 6 months. This was reflected in a low mean value (1.31), high right-skewness (3.59), and extremely high kurtosis (17.38).

Gaming problems were measured with the Ten-Item Internet Gaming Disorder Test (IGDT-10; Király et al., 2017). The psychometric properties of the IGDT-10 have been good in international studies (Király et al., 2019) and among Finnish vocational school students (Männikkö et al., 2019). The ten items measured, for example, gaming-related preoccupation, failure to moderate gaming, risking significant relationships, school, or work performance because of gaming, and playing despite negative consequences. The response choices were 0 (*never*), 1 (*sometimes*), and 2 (*often*). The participants gave their answers based on the previous 6 months here as well. Like the PGSI, we treated the IGDT-10 as a count variable, and the internal consistency of the scale was high (ω = 0.89). Similarly, 64.18% of the participants had not experienced any gaming problems within the last 6 months. Thus, the mean value for IGDT-10 was 1.34, skewness was 2.70, and kurtosis was 11.37.

The Basic Psychological Need Satisfaction and Frustration Scales (BPNSFS; Chen et al., 2015) were used to measure the satisfaction and frustration of basic psychological needs. The 24 items were translated to Finnish and back translated to check for accuracy. The participants evaluated how autonomy-, relatedness-, and competence-measuring claims applied to themselves on a seven-point Likert scale. For the purposes of this study, a need satisfaction scale and a need frustration scale were formed by combining three satisfaction subscales and three frustration subscales. The need satisfaction scale included items such as “I feel a sense of choice and freedom in the things I undertake” (autonomy satisfaction), “I feel that the people I care about also care about me” (relatedness satisfaction) and “I feel I can successfully complete difficult tasks” (competence satisfaction). In contrast, the need frustration scale included items such as “I feel pressured to do too many things” (autonomy frustration), “I feel excluded from the group I want to belong to” (relatedness frustration) and “I feel disappointed with many of my performances” (competence frustration). Both scales had very good internal consistency (ω = 0.93 for the need satisfaction scale; ω = 0.92 for the need frustration scale).

Mental health issues, gender, and age were used as control variables. Mental health issues were measured with the five-item Mental Health Inventory (MHI-5; Berwick et al., 1991), a shortened version of the original 38-item screen. MHI-5 has been used previously in multiple Finnish studies (Castrén et al., 2018, Elovanio et al., 2020, Salonen et al., 2020, Talala et al., 2008), and it is proven to be a valid and reliable instrument especially in associative studies (Elovanio et al., 2020). The five items measured mood, nervousness, sadness, calmness, and happiness (e.g., “How much of the time have you felt so down in the dumps that nothing could cheer you up?”) during the past month on a six-point Likert scale. Internal consistency of the measure was good (ω = 0.89). Descriptive statistics are summarized in Table 1.

### 2.3 Statistical analyses

Analyses were conducted with Stata 16 statistical software by StataCorp (2019). The Omegacoef package was used to calculate ω coefficients (Hayes & Coutts, 2020). Analyses do not include respondents who identified their gender as “other” (*n* = 4), so the sample size for analyses is 1526.

As both the PGSI and the IGDT had very high rates of zero-values and, consequently, postestimation for preliminary linear regression models showed a notable number of outliers, zero-inflated negative binomial regression (ZINB) with robust standard errors was applied to examine the associations between the independent and dependent variables. By dividing the analyses into two separate processes—count (susceptible group) and inflate (nonsusceptible group)—ZINB models can handle overdispersion and zero-inflation relatively well compared to other models (Yang et al., 2017). The use of zero-inflated models allowed us to examine the association of need frustration with the severity of gambling and gaming problems while examining the association of need satisfaction with inflated zeroes separately. Considering that the data should represent the general population, it can be assumed that not everyone is at risk of gambling or gaming problems. Thus, analyzing inflated zeroes separately should minimize possible biases that the inclusion of no-risk groups may bring into the models.

## 3 Results

As indicated by descriptive statistics (Table A.1), the distributions of gambling and gaming problems are extremely right-skewed and leptokurtic (slender-peaked). Out of participants, 427 (27.91%) reported having experienced at least one gambling-related risk (i.e., PGSI > 0) and 548 participants (35.82%) reported having experienced at least one gaming problem. The cut-off point for “problem gambling” (PGSI ≥ 8) was crossed for 93 participants (6.08%) and the cut-off point for “problem gaming” (IGDT-10 ≥ 5) was crossed for 165 participants (10.8%). Furthermore, correlation matrix (Table A.2) shows that nearly all variables were significantly correlated with each other. The only exception was male gender, which had only weak correlations with gambling and gaming problems, and mental health issues. The highest correlations were between gambling and gaming problems (0.53), need satisfaction and need frustration (-0.69), need satisfaction and mental health issues (-0.63), and need frustration and mental health issues (0.74). Gambling problems had a weak correlation to need satisfaction, need frustration, and mental health issues, while gaming problems were weakly correlated with need satisfaction but moderately correlated with need frustration and mental health issues. Age had a moderate inverse correlation with gaming problems, need frustration, and mental health issues, a weak correlation with need satisfaction and a weak inverse correlation with gambling problems.

**Table A1:** Descriptive statistics.

		Mean	SD	Range (Scale min–max)	Skewness	Kurtosis	ω
Gambling problems		1.31	3.33	0–25 (0–27)	3.59	17.38	0.95
Gaming problems		1.34	2.64	0–20 (0–20)	2.70	11.37	0.89
Basic psychological needs							
	*Satisfaction*	5.17	1.02	1–7 (1–7)	−0.48	3.03	0.93
	*Frustration*	2.75	1.13	1–6.67 (1–7)	0.47	2.70	0.92
Mental health issues		2.48	0.95	1–6 (1–6)	0.85	3.33	0.89
Age		46.67	16.42	18–75	−0.02	1.78	

**Categorical**		**N**	**%**				

Gender							
	*Male*	770	50.46				
	*Female*	756	49.54

**Table A2:** Correlation matrix.

	1.	2.	3.	4.	5.	6.	7.
1. Gambling problems	1						
2. Gaming problems	0.53***	1					
3. Need satisfaction	−0.21***	−0.26***	1				
4. Need frustration	0.23***	0.35***	−0.69***	1			
5. Mental health issues	0.23***	0.31***	−0.63***	0.74***	1		
6. Age	−0.16***	−0.32***	0.21***	−0.36***	−0.30***	1	
7. Male gender	0.11***	0.13***	−0.03	−0.03	−0.07*	−0.03	1

*p < 0.05; ** p < 0.01; *** p < 0.001.

In our first models (Table A.3), weaker mental health was significantly associated with increased gambling problems (IRR = 1.43, 95% CI 1.15; 1.78, *p <* 0.01) and gaming problems (IRR = 1.48, 95% CI 1.33; 1.64, *p* < 0.001), yet better mental health was associated only with the absence of gaming problems (i.e., inflated zeroes; OR = 0.68, 95% CI 0.55; 0.86 *p* < 0.001). Gender and age were significantly associated with the absence of gambling and gaming problems. Men and younger participants were less likely to report having no problems, while male gender and younger age were associated only with the severity of gaming problems.

**Table A3:** Zero-inflated negative binomial regression models for gambling and gaming problems.

	**Gambling problems**				**Gaming problems**			
	IRR	Robust SE	95% CI		IRR	Robust SE	95% CI	
Mental health issues	1.43**	0.16	1.15	1.78	1.48***	0.80	1.33	1.64
Male gender	1.42	0.35	0.87	2.30	1.48***	0.16	1.19	1.83
Age	0.99	0.01	0.98	1.00	0.99**	0.00	0.98	1.00

***Inflated zeroes***	**OR**	**Robust SE**	**95% CI**		**OR**	**Robust SE**	**95% CI**	

Mental health issues	0.43	0.53	0.04	4.73	0.68***	0.08	0.55	0.86
Male gender	0.46*	0.14	0.26	0.83	0.61*	0.12	0.42	0.89
Age	1.03*	0.01	1.00	1.05	1.05***	0.01	1.04	1.07

Wald χ2: (3)	34.23				73.72			
Max. likelihood R2	0.09				0.21			
Cragg & Uhler’s R2	0.10				0.23			
McFadden’s Adj. R2	0.03				0.08			

*p < 0.05; ** p < 0.01; *** p < 0.001

Our second models (Table A.4) are similar to the previous models, but with the addition of basic psychological need satisfaction and frustration scales. While mental health issues were significantly associated with both gambling and gaming problems in the previous models, this association became nonsignificant when need frustration was added to the models. Need frustration, in turn, was positively associated with the severity of both gambling problems (1.22, 95% CI 1.03; 1.43 *p* < 0.05) and gaming problems (1.34, 95% CI 1.21; 1.49 *p* < 0.001). However, better mental health still had a similarly strong association with the absence of gaming problems after the addition of need satisfaction. In fact, need satisfaction had no significant association with the absence of gambling or gaming problems. Moreover, gender and age were still associated with the absence of gambling and gaming problems and with the severity of gaming problems.

**Table A4:** Zero-inflated negative binomial regression models for gambling and gaming problems. Need frustration on the upper (count) section, need satisfaction on the lower (inflate) section.

	Gambling problems				Gaming problems			
	IRR	Robust SE	95% CI		IRR	Robust SE	95% CI	
Need frustration	1.22*	0.10	1.03	1.43	1.34***	0.07	1.21	1.49
Mental health issues	1.25	0.21	0.89	1.75	1.12	0.08	0.96	1.29
Male gender	1.31	0.31	0.82	2.10	1.47***	0.16	1.19	1.82
Age	0.99	0.01	0.98	1.00	0.99*	0.00	0.99	1.00

***Inflated zeroes***	**OR**	**Robust SE**	**95% CI**		**OR**	**Robust SE**	**95% CI**	

Need satisfaction	1.06	0.21	0.72	1.56	0.97	0.10	0.79	1.20
Mental health issues	0.58	0.53	0.10	3.45	0.65**	0.10	0.48	0.87
Male gender	0.47**	0.12	0.28	0.78	0.61**	0.12	0.42	0.89
Age	1.02*	0.01	1.00	1.05	1.05***	0.01	1.04	1.07

Wald χ2: (4)	34.20				123.59			
Max. likelihood R2	0.09				0.23			
Cragg & Uhler’s R2	0.10				0.24			
McFadden’s Adj. R2	0.03				0.08			

*p < 0.05; ** p < 0.01; *** p < 0.001

We also tested for the interaction between mental health issues and basic psychological need satisfaction/frustration but found that the interactions were not statistically significant. The only independent variable that held statistical significance was need frustration when gaming problems were the dependent variable (1.64, 95% CI 1.20; 2.26 *p* < 0.01), but as the interaction term was not significant, this association also held little practical significance.

## 4 Discussion

This study focused on the associations between satisfaction and frustration of basic psychological needs and gambling and gaming problems. We found that need frustration was associated with both gambling and gaming problems. When gambling or gaming problems were present, higher need frustration was associated with increases in their severity. Mental health issues had a significant association with the severity of gambling and gaming problems only before basic psychological need frustration was added to the models. However, our second hypothesis was disputed, as need satisfaction was not associated with the absence of either of these problems, while better mental health was only associated with the absence of gaming problems. The relationship between mental health issues and the absence of gaming problems was inverse, meaning that the likelihood of having any gaming problems increases when mental health issues increase. Finally, our third hypothesis was disputed, as there were no significant interactions between mental health issues and need satisfaction or frustration.

Based on our results, gambling and gaming problems seem to be quite similar in regard to the frustration of basic psychological needs. Moreover, gender and age are similarly associated with absence but not with the severity of these problems. This suggests that men and younger people are more likely to develop at least some problems related to their gambling or gaming, but once problems exist, these same factors matter only for the severity of gaming problems. Our results also suggest that mental health issues increase the risk of having at least some gaming problems, but contrary to previous knowledge (APA, 2013; Kessler et al., 2008, Petry et al., 2005), the same was not true for gambling problems. However, mental health issues did have a significant association to gambling problems before the addition of basic psychological need frustration, and even while we found no significant interactions between these factors, the correlation matrix revealed a high correlation between them. Therefore, a possibility remains that there is a more complex interaction between the variables that we did not account for here. Further research could delve deeper into these complex dynamics between mental health, need frustration, and gambling or gaming problems.

According to the SDT, frustration of basic psychological needs can make the frustrated individual turn to maladaptive substitute behaviors to gain at least momentary satisfaction (Ryan and Deci, 2000, Ryan and Deci, 2017, Vansteenkiste and Ryan, 2013). As gambling and digital gaming are pleasurable activities, with excitement related to their promises of winnable challenges (Binde, 2013, Boyle et al., 2011) it is understandable to seek a sense of satisfaction next to them. Following the SDT, need frustration relates to more problematic forms of gambling and gaming, which was our first hypothesis. Our models supported this hypothesis, as higher need frustration was associated with more severe gambling and gaming problems. This finding also supports previous studies that have found that need frustration is a mediating factor in the effects of motivation and amotivation on gambling problems and psychological distress and may increase gaming problems by lowering self-control (Mills et al., 2021, Mills and Allen, 2020).

Our second hypothesis about the satisfaction of basic psychological needs being associated with the absence of gambling and gaming problems was not supported by our models. This is surprising, considering how previous studies have shown that need satisfaction may protect against the risk of developing gambling or gaming problems (Allen and Anderson, 2018, Dennis et al., 2017, Tóth-Király et al., 2019). Having a nonsignificant association between these problems and need satisfaction indicates that this might not be the case, at least at the general Finnish population level. One obvious explanation for this could be that in a country like Finland, where gambling and gaming are widespread and visible activities, people might still experience some gambling- or gaming-related problems even with high need satisfaction. This should be investigated further in future research.

Furthermore, our additional analyses on interactions between need satisfaction/frustration and mental health issues did not seem to support our third hypothesis, as the interaction term was not significant. While it is likely that some covariance exists, considering the high correlations between these variables (see Table 1), VIF tests showed that multicollinearity was not a problem. Therefore, it is reasonable to conclude that need satisfaction and frustration can be considered independently associated with the severity of gambling and gaming problems when mental health issues are controlled.

The current study includes some important limitations. First, response rate for the survey was 34.6% and limited to panel participants, which may introduce potential self-selection bias. For example, it is possible that our participants found the topic of the survey more interesting than those who did not respond to the survey. Moreover, our sample includes slightly more gamblers than some population estimates (Salonen et al., 2020). However, considering the aims of the study, this is not a limitation, as our analyses are robust, and the data is demographically balanced. Due to the cross-sectional study design, no causal assumptions can be based on these results. While it can be assumed that the frustration of basic psychological needs or problems with mental health can lead to gambling or gaming problems, the reverse assumption is equally plausible. The survey was also based on self-reports, which means the measures are more subjective and influenced by interpretations that the participants might have of their situation. Design-wise, we did not analyze each basic psychological need separately, as our focus was on their combined association with gambling and gaming problems.

All in all, our findings add to the growing number of studies that approach gambling and gaming problems from the perspective of satisfaction and frustration of basic psychological needs. Further research is needed to study the effects of autonomy, relatedness, and competence separately to learn about their possible individual relationships to these problems and whether there are similarities or differences in these associations. More importantly, longitudinal designs could clarify the cause-and-effect relationships between basic psychological needs and gambling and gaming problems to see in more detail how they influence each other.

### 4.1 Conclusions

Basic psychological needs are considered necessary factors in well-being. This study approached gambling and gaming problems as possible results of low need satisfaction and high need frustration. While need satisfaction was not associated with the absence of these problems, need frustration was associated with the severity of both gambling and gaming problems. Theoretically, our results are very much in line with other studies testing the premises of SDT and suggest that problems related to gambling and gaming coexist with frustration of basic psychological needs. It would thus be worthwhile to consider how these needs are fulfilled in the daily lives of those who experience these problems when they seek support and treatment. More importantly, these results highlight the importance of (preferably society-wide) need supportive environments in management of problems that might accompany behaviors such as gambling and gaming.

**Funding sources**

The study was funded by the Finnish Foundation for Alcohol Studies (Gambling in the Digital Age Project, 2021–2023, PI: A. Oksanen). Two of the authors received personal grants: Ilkka Vuorinen was supported by a grant from the Jenny and Antti Wihuri Foundation and Heli Hagfors by a grant from the Finnish Foundation for Alcohol Studies.

#### CRediT authorship contribution statement

**Ilkka Vuorinen:** Conceptualization, Data curation, Formal analysis, Funding acquisition, Investigation, Methodology, Resources, Software, Validation, Visualization, Writing – original draft, Writing – review & editing. **Iina Savolainen:** Conceptualization, Data curation, Funding acquisition, Investigation, Resources, Writing – original draft, Writing – review & editing. **Heli Hagfors:** Conceptualization, Funding acquisition, Investigation, Resources, Writing – original draft, Writing – review & editing. **Atte Oksanen:** Conceptualization, Data curation, Funding acquisition, Investigation, Methodology, Project administration, Resources, Software, Supervision, Writing – original draft, Writing – review & editing.

## Declaration of Competing Interest

The authors declare that they have no known competing financial interests or personal relationships that could have appeared to influence the work reported in this paper.
